# Reinforcement of Environmental DNA Based Methods (*Sensu Stricto*) in Biodiversity Monitoring and Conservation: A Review

**DOI:** 10.3390/biology10121223

**Published:** 2021-11-23

**Authors:** Pritam Banerjee, Gobinda Dey, Caterina M. Antognazza, Raju Kumar Sharma, Jyoti Prakash Maity, Michael W. Y. Chan, Yi-Hsun Huang, Pin-Yun Lin, Hung-Chun Chao, Chung-Ming Lu, Chien-Yen Chen

**Affiliations:** 1 Department of Biomedical Science, Graduate Institute of Molecular Biology, National Chung Cheng University, 168 University Road, Min-Hsiung, Chiayi County, Jiayi 62102, Taiwan; pritam8683@gmail.com (P.B.); dgobinda1993@gmail.com (G.D.); chanmwy@gmail.com (M.W.Y.C.); 2 Department of Earth and Environmental Sciences, National Chung Cheng University, 168 University Road, Min-Hsiung, Chiayi County, Jiayi 62102, Taiwan; raju28212@gmail.com (R.K.S.); jyoti_maity@yahoo.com (J.P.M.); x699237014x@gmail.com (Y.-H.H.); ekman60@gmail.com (H.-C.C.); 3 Department of Theoretical and Applied Science, University of Insubria, Via J.H. Dunant, 3, 21100 Varese, Italy; cm.antognazza@gmail.com; 4 Department of Chemistry and Biochemistry, National Chung Cheng University, 168 University Road, Min-Hsiung, Chiayi County, Jiayi 62102, Taiwan; kittycat1604@hotmail.com; 5 Department of Chemistry, School of Applied Sciences, KIIT Deemed to be University, Bhubaneswar 751024, India; 6 Department of Chemical Engineering, National Chung Cheng University, 168 University Road, Ming-Shung, Chiayi County, Jiayi 62102, Taiwan; n0935718023@gmail.com

**Keywords:** environmental DNA application, biodiversity monitoring, invasive species, species under conservation, molecular ecology, conservation management

## Abstract

**Simple Summary:**

Worldwide biodiversity loss points to a necessity of upgrading to a fast and effective monitoring method that can provide quick conservation action. Newly developed environmental DNA (eDNA) based method found to be more cost-effective, non-invasive, quick, and accurate than traditional monitoring (spot identification, camera trapping). Although the eDNA based methods are proliferating rapidly, as a newly developed branch, it needs more standardization and practitioner adaptation. The present study aims to evaluate the eDNA based methods, and their potential achievements in biodiversity monitoring, and conservation for quick practitioners’ adaption. The investigation shows that the eDNA technique is applicable largely in (i) early detection of invasive species, (ii) species detection for conservation, (iii) community-level biodiversity monitoring, (iv) ecosystem health monitoring, (v) study on trophic interactions, etc. Thus, the eDNA technique shows a great promise with its high accuracy and authenticity, and will be applicable alone or alongside other methods in the near future.

**Abstract:**

Recently developed non-invasive environmental DNA-based (eDNA) techniques have enlightened modern conservation biology, propelling the monitoring/management of natural populations to a more effective and efficient approach, compared to traditional surveys. However, due to rapid-expansion of eDNA, confusion in terminology and collection/analytical pipelines can potentially jeopardize research progression, methodological standardization, and practitioner adoption in several ways. Present investigation reflects the developmental progress of eDNA (*sensu stricto*) including highlighting the successful case studies in conservation management. The eDNA technique is successfully relevant in several areas of conservation research (invasive/conserve species detection) with a high accuracy and authentication, which gradually upgrading modern conservation approaches. The eDNA technique related bioinformatics (e.g., taxon-specific-primers MiFish, MiBird, etc.), sample-dependent methodology, and advancement of sequencing technology (e.g., oxford-nanopore-sequencing) are helping in research progress. The investigation shows that the eDNA technique is applicable largely in (i) early detection of invasive species, (ii) species detection for conservation, (iii) community level biodiversity monitoring, (iv) ecosystem health monitoring, (v) study on trophic interactions, etc. Thus, the eDNA technique with a high accuracy and authentication can be applicable alone or coupled with traditional surveys in conservation biology. However, a comprehensive eDNA-based monitoring program (ecosystem modeling and function) is essential on a global scale for future management decisions.

## 1. Introduction

The loss of biodiversity has been one of the most serious concerns worldwide. The world has been losing its biodiversity due to a target to fulfilling high demands of satisfaction by the human race which in turn is incurring expensive and detrimental demands to nature [1]. According to IPBES (The Intergovernmental Science-Policy Platform on Biodiversity and Ecosystem Services) report, 25% of animals and plants are already threatened with extinction [2]. The wild animals and plants, as well as domestic ones, are facing a fight for survival due to anthropogenic activity. In the next thirty years, 30–50 % of plant species will become extinct [3]. The current rate of extinction is 1000 to 10,000 times greater than the natural extinction rate on our planet [2]. This is an extremely serious issue that will be more severe in the coming days. However, to mitigate this issue, we need to initiate a monitoring program at both local and global levels. In designing such a monitoring system, we need to consider the development of a fit-for-purpose, accurate and cost-effective technique for the detection of species, assessment of biodiversity and study of species interactions.

**Figure 1:**
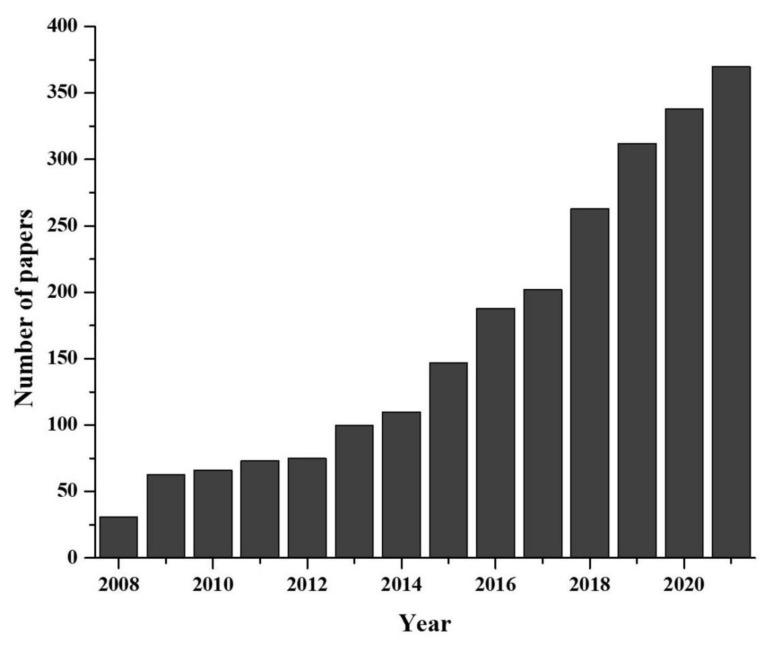
Developmental progress of eDNA technique in last two decades (data collected from PUBMED advanced search with “environmental DNA or eDNA” as title).

Environmental DNA, known as eDNA, is shed by organisms during their existence in nature [4]. During their lifespan, organism shed DNA wherever they have been present for a moments. The collection and analysis of these environmental samples and monitoring of the ecosystem without harming organisms is the basis of eDNA study. Recently eDNA, provided valuable contribution to both aquatic and terrestrial monitoring [5,6]. Originally, the eDNA-based species detection was a microbiological study, dating back in 1987 [7] and the use of eDNA to detect macro-organism directly from water sample came to the front in early 2008 with detection of aquatic invasive species [8]. Later on, the methodology was updated and reinforced by some pioneer studies [9,10]. Afterward, rate of eDNA release, degradation, persistence as well as the changes in concentration with organism abundance were explored [11,12,13,14,15,16]. However, as more studies incorporated the use of eDNA approaches, terminology quickly diverged, becoming more convoluted or generally misunderstood [17,18]. What quickly followed was two distinct schools of thought: (i) those who view eDNA as relating to any DNA originating from environmental samples (eDNA *sensu lato*: [4]), and (ii) research referring to eDNA originating from macro-organisms specifically (eDNA *sensu stricto*). Researchers use eDNA for species detection to reveal many critical ecological questions, such as studies of population genetics, abundance and habitat preference, detection of unrecorded populations, understanding behavioral biology, monitoring of reproductive migration, pathogens, terrestrial plant community, biodiversity of marine and river ecosystem, nutrient quality, assessment of coral ecosystem, etc. [19]. Moreover, the novel eDNA-based approaches have been used to solve some critical conservation issues such as the detection of rare and endangered species [20], invasive species [21], monitoring whole biodiversity [22,23], study of anthropogenic effect [24], ecosystem health [25] and disease [26]. As eDNA-based methods are emerging rapidly as a multidisciplinary branch of science (Figure 1), it is necessary to evaluate the recent advancements for proper implementation.

Considering this background, the aim of the present investigation is to focus on and summarize the methodological development and application of macro-organismal eDNA in biodiversity monitoring, highlighting successful case studies in conservation management. By doing so, we hope to draw the attention of practitioners who may otherwise be unfamiliar with the achievements of eDNA-based methods that have been made to date.

## 2. Overview of Sampling and Laboratory Protocol for eDNA

The detection of biological signature from eDNA traces has been reported from different environment (e.g., water, soil, air, snow, and even in drinking water) [27], where the sampling approaches and extracting protocols of eDNA have indicated ‘required modifications’ depending on sample type and interest [16].

### 2.1. Collection and Accumulation of eDNA Samples

The long-time exposure and abundance of target organisms strongly increase the amount of eDNA into the environment [28], where detection probability was reported to be higher near the habitat [29]. Nevertheless, organisms in low abundance can be traced with meticulous experimental design [30]. The occurrence of the targeted eDNA in the environment depends upon their life history, body size, behavior, seasonal and reproductive activity (e.g., eDNA amount observed is higher during the breeding season) [6]. The persistence may depend upon the physicochemical factors of the environment (e.g., temperature, pH, and oxygen) [29]. Not only the physicochemical characteristics of the medium, but also the mobilization of the medium (e.g., intra-medium: water to water; inter-medium: soil to water) influences the existence/persistence of eDNA in the environment, including false-positive detection due to multiple factors (e.g., medium current, settlement, predator, anthropogenic activity, etc.) [28].

#### 2.1.1. Aquatic Environment

Generally, in aquatic biomes, the existence of eDNA differs according to sampling zones (e.g., littoral, limnetic, and intertidal) [6,16]. The different effective samplers (e.g., Nansen metal water sampler, Bucket and van Dorn sampler, Kemmerer type water sampler, Niskin water sampler, Bottles, PVC pole, and Polyethylene Nalgene bottles, etc.) are used in different studies for eDNA sampling [31,32]. In the case of lentic ecosystems, eDNA deposits in sediments, since water is stagnant, whereas long-dated eDNA is observed in significantly higher amounts in sediment zone compared to photic zone [33]. Here, a well cleaned DNA free bottle or one-time use sampler is suitable for the collection, whereas a sampler equipped with pole/rope-like structure is used for benthic water sampling [34]. On the other hand, in the lotic ecosystem, a filter funnel can be used against the flow to collect eDNA from water; however, the chances of false-positive results (due to transportation of eDNA) should be kept in consideration.

The sample can be processed through filtration/centrifugation/ultra-centrifugation/ precipitation steps after collection (if the accumulation step is not performed) [32]. The filtration technique (as it processes larger volumes of water at once) is the most common method adopted for accumulation of DNA into filter paper (0.2–3.0 µm size) [32,35]. The main problem of this technique is clogging, in particular for smaller pore filters; here the use of two or more separate filter papers for samples can be adopted. Moreover, conventional filtration, enclosed sterivex filters (pressure mediated filter without electricity) are highly effective and advanced techniques in remote field surveys [35]. The ethanol precipitation, centrifugation, and ultra-centrifugation steps are suitable for places where long-time access is difficult and when the targeted DNA is present in high concentration (because those generally process less volume of sample) [32,35]. In the precipitation step, ethanol or isopropanol are required to accumulate DNA; however, the centrifugation and ultra-centrifugation steps require no chemicals [16]. Furthermore, the detection probability is correlated with sample volume, although it is necessary to optimize it depending on target organism [36]. Tsuji et al. [32] reviewed the protocols on eDNA studies in water, which indicated that over 78% of cases used the filtration method, followed by ethanol precipitation (13%) and centrifugation (4%). The most common type of filter paper is used as cellulose nitrate (CN) 0.45 µm pore size, although other types (0.45 µm mixed cellulose ester membrane, 0.7 µm glass microfiber, etc.) are also considered [32,37].

#### 2.1.2. Terrestrial Environment

The selection of different soil layers eventually depends on targeted taxa. The soil may be collected using a sterile digger or debris metal screens (to remove large particles), and collected soil needs to be kept in a dark box (containing ice) for the transpiration purpose to the laboratory as soon as possible for DNA extraction [38]. In another technique, the soil sample can be dissolved in water by agitation, followed by filtration (like water) to concentrate the DNA into filter paper. The sterile tubes, modified plastic syringes, and drilling core samples can be inserted into the sediment to withdraw the sediment samples for eDNA study. In the case of air sampling, especially designed volumetric samplers can be used [23].

#### 2.1.3. Extraction of eDNA from Other Organisms without Isolating Target Taxa

The eDNA also can be extracted from a non-target organism to study species interaction data of target organisms, such as feces to study the dietary information, insect-derived DNA (iDNA) to study mammalian diversity, flower to study plant-pollinators-interaction, water from sponges to study marine diversity, etc. [39,40,41,42].

### 2.2. Preservation of Samples in eDNA Technique

The quality and quantity of DNA undergoes degradation/change due to microbial activity, mechanical forces, chemical reactions, etc., hence the preservation of sample is an essential step for experimental design [37]. On-site, if filtration and/or preservation is not possible then samples should be stored in dry-ice or in a styrofoam box with cooling elements (just after collection) and at −20 °C in the laboratory (not more than 8 h) [32,37]. Dried or semi-dried samples (e.g., soil, feces) also can be stored in a pouch containing silica beads. The filter is generally preserved by freezing in a liquid medium or dry medium. The liquid preservative (e.g., ethanol, Longmire’s buffers, cetyl trimethyl ammonium bromide (CTAB), ATL lysis buffer (Qiagen, Hilden, Germany), etc.) are effective to store DNA present on the filter paper [32,37]. Alternatively, filter paper (wrapped in aluminum foil or directly) can be placed in a silica gel containing packet (to keep it moisture free) [37].

### 2.3. The eDNA Analysis in Laboratory

Samples should be stored in dry preservative, which later on can undergo extraction process directly (e.g., soil sample) [43], whereas the filter paper should be stored in ethanol or other liquid media, before DNA extraction, and should be kept in an open micro-centrifuge tube under a fume hood to let the liquid evaporate properly. On the other hand, for centrifugation, ultra-centrifugation, and precipitation steps, DNA can be extracted from pellets [37]. It is important to mention that both conventional (e.g., CTAB) and commercial kits (time-saving and less hazardous) are available (e.g., DNeasy blood tissue kit, Qiagen) for DNA extraction [32,37].

In the case of single species detection, eDNA is subjected to amplification with species-specific primer [44]. Here, conventional PCR (cPCR) can be used to conduct a ‘presence and absence’ study whereas quantitative PCR (qPCR) is more preferred for quantification of targeted DNA and elimination of false positive or negative results [16,45]. In qPCR, probes are one of the best option to identify particular species, although intercalating dye (e.g., SYBR Green) can be used instead of probes for cost-effectiveness [45]. However, the droplet digital PCR (ddPCR) (sensitive PCR) has better species specificity and quantification accuracy than the formers [31]. Recently, CRISPR-Cas is gaining popularity in eDNA-based species detection [46]. Moreover, in all cases, positive and negative controls should be maintained [44].

**Figure 2:**
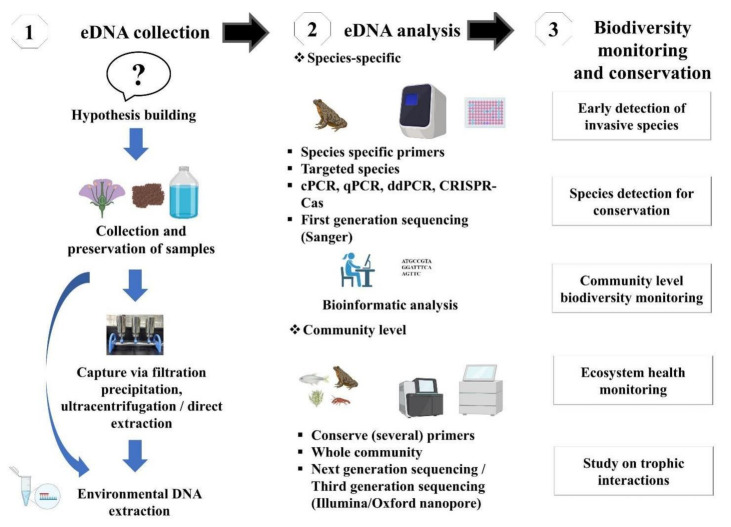
Schematic workflow of eDNA-based studies and its application in biodiversity monitoring and conservation.

The evolution of technology has allowed to introduce a new high-throughput sequencing (HTS) platform enabling analysis and identification of whole communities, commonly termed DNA metabarcoding [4]. The HTS platform can produce billions of sequences in a single run, allowing analyzing several samples in parallel and identifying several species in each sample. Such an advancement leads to an increase in the computational load, and it is imperative to move toward high-performance computing. Furthermore, DNA metabarcoding is a widely tested and validated approach for processing mixed taxon. The community detection through DNA metabarcoding relies on “universal” primers (i.e., non-specific primers), therefore introducing amplification bias when the primers match some taxa better than others during PCR amplification [38]. This bias might be counteracted using hybridization probes by allowing the targeted capture of barcoding genes. An alternative is to sequence directly the extracted bulk DNA without PCR [47]. These metagenomic techniques overlook most of the problems associated with PCR-based metabarcoding, such as the loss of some taxonomic groups due to primer binding sites [48], and they are well established for bacterial communities and recently applied to eukaryotes [38]. An advisable procedure is to design a metabarcoding-based study in order to be ecosystem-specific and target-gene primer sets. Considering the desired ecosystem and taxonomic context, in-silico and in-vitro tests should be performed to validate the applicability of the primer pairs. PCR primers (species-specific or universal) can be designed manually or using software (e.g., Primer Express 3.0, 3.0.1, NCBI primer blast, allele ID) [48]. Furthermore, practitioners can also consult for standard manual or technical advice to eDNA societies/private sectors, such as DNAqua-Net (https://dnaqua.net/), EnviroDNA (https://www.envirodna.com/), CaleDNA (https://ucedna.com/), The eDNA society, Japan (https://ednasociety.org/en/), etc., and all of the links are accessed on 20 November 2021. Moreover, a general outline of eDNA based technique is presented in Figure 2.

## 3. Precautions in eDNA Study

Contamination can arise from various sources (indicated below) at any stage during the sampling and laboratory analysis which can distort the result precision.

### 3.1. Precautions in Field

In the field, contamination is a major issue for inter and intra sampling. The sterilized sampling boots or sterile chest waders should be used when the researcher is required to reach deep into the sampling site for sample collection [44]. It is highly recommended that all tools should be sterilized with 10–50% bleach solution followed by deionized water (>2 times) [30]. Special care should be taken during opening the filter paper (sterile filters should be preferred) from the package and after filtration, as filter paper should be removed using previously sterilized forceps.

### 3.2. Precautions in Laboratory

In the laboratory, although a specific or universal primer is present, there is a chance of false-positive or false-negative detection. Types of errors in DNA-based detection are well-reviewed by Darling et al. [49]. To obtain accurate results in the laboratory, there are several precautions that should be taken under consideration: (i) cleanliness, (ii) wearing of clean clothes, (iii) one-time use of gloves and a facial masks, (iv) cleaning of the surface of workplace using chemical (e.g., DNA Away, Decon 90, DNA-exitusPlus and bleach solution) and physical methods (UV light), (v) DNA extraction in a contamination-free zone (vi) restriction of movement during while handling PCR, (vii) use of proper control samples, and (viii) totally unidirectional workflow, [30,44].

## 4. Application of eDNA in Conservation Biology

The biodiversity monitoring implies multiple aspects to be studied and understood from species distributions, interactions, abundance, invasiveness to ecosystem health, imbalance, and climatic effects. The ecosystems need a frequent and accurate monitoring program due to the large decline in biodiversity. The proper management of biodiversity (by a suitable monitoring method) protects the ecosystem-change due to anthropogenic activity [1], but delay in understanding their effect can cause ecological and economic loss. Indeed, eDNA-based monitoring can provide ecological data for taxon including presence/absence, abundance, habitat dispersion, immigration, emigration, community interaction, and species distribution [38]. eDNA can be applied in regular monitoring programs in zoological parks, botanical parks, national parks, and protected areas [50].

### 4.1. Early Detection of Invasive Species

The invasive species are the ‘introduced species’ into a new place (naturally or anthropogenically) where they begin to proliferate rapidly and outcompete native species [51,52], leading to destruction of resources while also carrying new pathogen strains into introduced places [51]. Regular basis monitoring and documenting their distribution is needed for accurate conservation management. Pioneer studies on eDNA-based work reported the detection of invasive American bullfrog (*Lithobates catesbeianus*) in aquariums and freshwater systems [8]. Thereafter, a progression of interest in eDNA-based study on invasive species was noticed. Now, the feasible success of eDNA-based monitoring is not only limited to small freshwater species, but also to large semi-aquatic macro-organism (e.g., *Python bivittatus*), and terrestrial mammals (*Sus scrofa*) [34,53,54]. Till now, several invasive species throughout all taxonomic groups such as fish (e.g., *Hypophthalmichthys nobilis*, *Salvelinus fontinalis*, *Oncorhynchus mykiss*, *Esox lucius*, *Salmo trutta* etc.), amphibian (e.g., *Lithobates catesbeianus*), arthropod (e.g., *Aedes albopictus*, *Eriocheir sinensis*, *Rhithropanopeus harrisii*, *Crangonyx pseudogracilis*), Mollusca (e.g., *Dreissena polymorpha*, *Limnoperna fortune*, *Crepidula fornicate* etc.), Reptile (e.g., *Python bivittatus*, *Trachemys scripta* etc.), mammals (e.g., *Sus scrofa*), angiosperms (e.g., *Elodea canadensis*), etc. are detected from diverse environment (freshwater, seawater, etc.) efficiently by eDNA-based method (Table 1). Although progress in methodological standardization for detection of invasive macro-organism from terrestrial habitat (soil, air) is under study [55].

### 4.2. Species Detection for Conservation

A species under conservation needs regular basis monitoring and management [30]. If the species present in the environment is low in abundance, over time it leads to them being endangered, mostly due to anthropogenic activity, and sometimes naturally. Application of eDNA based investigation on critically endangered species is not limited to a single taxon but also for detecting multiple taxa at a time through eDNA metabarcoding [78,79], with advantages present such as short-term monitoring and cost-effectiveness. The detection of species under conservation (e.g., rare, indicator, endangered, vulnerability, etc.) and their present conservation status in different environments, including their barcoding regions by eDNA, are shown in Table 2, where it has been noted that eDNA has successfully adapted standardization and is gradually growing to be an effective monitoring system. Monitoring of different taxa under conservation, such as Arthropod (e.g., *Cambarus speleocoopi*; Endangered, *Pacifastacus fortis*; Critically Endangered; *Baetis buceratus*; Vulnerable) Amphibia (e.g., *Triturus cristatus*; Lest Concern, *Odorrana splendida*; Endangered, *Cryptobranchus alleganiensis*; Near Threatened), Fish (e.g., *Spirinchus lanceolatus*; Threatened, *Hypophthalmichthys molitrix*; Near Threatened, *Salvelinus confluentus*; Vulnerable, *Misgurnus fossilis*; Least Concern), Reptile (e.g., *Shinisaurus crocodilurus*; Near Threatened), Mammals (e.g., *Neophocaena asiaeorientalis*; Endangered) and Angiosperm (*Sapria himalayana*; Endangered) signifies that performance of eDNA remains same irrespective of different environments (lake, stream, pond, soil), where variation in methods aid to a successful detection. However, sometimes, the eDNA technique can suffer from low detection probability [e.g., detection of the critically endangered animal, large-tooth sawfish (*Pristis pristis*)] [78]. Still, in most cases (as noted in Table 2) the eDNA method successfully detects species under conservation.

### 4.3. Biodiversity Monitoring at the Community Level

Advantages over conventional monitoring in short-time detection of biodiversity helps eDNA concept to proliferate. Pioneer studies began in 2012 [13,14] on detection of rare and endangered species in freshwater, and successfully demonstrated the potential of eDNA metabarcoding in ecosystem monitoring. In one study, the earthworm communities and patterns of plant taxonomical diversity was estimated by DNA-based monitoring (based on extracellular soil DNA) [102]. In the marine environment, the eDNA metabarcoding was reported by Thomsen et al. [13], where 15 different fish species, one rare species (*European pilchard*), and four bird species were successfully detected. Drummond et al. [43] critically evaluated the metabarcoding approach of eDNA in eukaryotic biodiversity assessment where a broad diversity of eukaryotes was identified from soil DNA. Deiner et al. [5] advocated the useful way of eDNA to uncover biodiversity information, they detected 296 families of eukaryotes from aquatic ecosystems, and interestingly they also detected signals from terrestrial life, which indicates the transport of DNA across the environment. Later, eDNA collected from the stream was noted to identify the same mammals with camera trapping methods, and this also suggests the possible detection of terrestrial life from its near aquatic regions. Furthermore, the eDNA method was also successfully used in the detection of terrestrial plant communities, fungi in the soil, arthropods, other invertebrates as well as large terrestrial mammals (Table 3). Ushio et al. [50] developed a method for the successful detection of avian species with Mibird primer, and clearly addressed that eDNA-based monitoring is not restricted to a particular group, but also to different taxonomic groups in the environment. The wetland biodiversity was explored by Shackleton et al. [103], considering landscape patterns with community diversity (both aquatic and terrestrial life). The recent advancement of eDNA metabarcoding methods improved the understanding of migratory biodiversity, which was a critical task in conservation biology [94]. Furthermore, the indirect extraction of DNA (DNA from a non-targeted organism) also has the potentiality to give a snap-shot of the community (or a taxonomic group) (e.g., metabarcoding of wildflowers came with animal-interaction in nature: arthropods diversity) [39]. Additionally, eDNA metabarcoding also allows us to figure out the trophic interaction relationships using the DNA from stomach content, feces samples, and even from the surface of organisms etc. [39,104,105,106]. Thus, eDNA-based methods provide information about trophic level interactions which is strongly needed for understanding more complex ecology where conventional methods failed. Furthermore, the monitoring of ecosystem health is also a critical concern in conservation biology, with the strong impact of anthropogenic activity, biological invasion, risk of epidemics, etc. The eDNA method is also applicable in disease monitoring in ecosystem health [26], pathogenic organisms [25,107] as well as anthropogenic impact in disbalancing ecosystem health due to pollution, global warming, deforestation, etc., [108].

## 5. Current Research Focus

Even though the eDNA technique has proven to be a useful emerging method in biomonitoring and conservation research, there is a need to understand the current research breaches for its upgradation, like; (i) persistence of eDNA in the environment, as well as understanding the age of eDNA, where current work is going on (see also, Marshall et al. [116]), (ii) lack of understanding in eDNA ecology (see also, Rodriguez-Ezpeleta et al. [18]), where further is study needed (iii) field and laboratory standardization, where most of the practitioners face problems, (for details see some standard protocols e.g., ‘The eDNA society’, Japan, https://ednasociety.org/en/, accessed on 20 November 2021) (iv) population genetic data through eDNA, where recent study came with allelic frequency from eDNA [117], (v) employment of eDNA beyond aquatic environment where recent research reveals that animal eDNA can be isolated from the air [118], (vi) comparison with conventional monitoring to make eDNA more authentic, and (vii) the improvement of bioinformatic studies, etc. These are a few of the main focuses in the coming years for developing eDNA as a global monitoring method.

## 6. Recommendation of eDNA Study and Future Perspective

Through the rapid development of technology, the eDNA-based methodologies have been proven to be highly successful for surveying species-community and monitoring biodiversity. The ecosystem is a complex system, made of a great number of species with biotic and abiotic interactions, where the conventional techniques are not powerful enough. Presently, next-generation biomonitoring based on eDNA offers great opportunities: the amount of data it can generate, which will allow researchers to address the fundamental ecological question, such as (i) which species coexist in given ecosystems, and (ii) how they interact and shape the ecosystem in space and time [119]. Nevertheless, current assessments of ecological quality would need to be adapted to the eDNA metabarcoding framework, to allow this technique to truly achieve the best of its potential. Models and ecological concepts need to be adapted in ways that allow the use of presence/absence data and non-traditional abundant/biomass estimates at least until the issue of molecular quantification of these parameters have been resolved and standardized [120]. It will be important that all these changes will be feasible on a large scale, particularly when considering thresholds, internationally, and the differences between traditional and molecular methods: the aim would be standardization across nations and researchers [121]. In this way, the scientific community would reach a comprehensive eDNA-based monitoring program, possibly in a few years, across a variety of taxa and environments, allowing providing a framework, on a global scale, for both ecosystem modeling and function with the ultimate goal of informing future management decisions [122]. Even though in the last decade huge efforts have been conducted in place to increment data repository, a critical step in the following years to further expanding reference databases - aiming to include identifiable sequences for all target biodiversity [122]. Furthermore, optimization of bioinformatics pathways with also the development of user-friendly interfaces would contribute to improve a wide-spread implementation [80]. Indeed, there is an increase of researchers, industry and governments incorporating eDNA survey into their toolkit for bio-surveys due to (i) ethical reasons, (ii) high accuracy, (iii) cost-effectiveness, (iv) safety, (v) inaccessible environments, and even (vi) by non-experts [122]. The biodiversity assessments should be rapid, cost-effective, and non-invasive, which are important in conservation biology [22]. Even though automation of eDNA sampling seems not far away, further research is needed around the fate of eDNA in all ecosystems, to understand the temporal longevity and spatial dispersal in order to consider fully valid studies about abundance and richness [22,121]. However, detection of false positive and negative detections is still a matter of concern as it may generate false biodiversity information, such as false detection of endangered species may manipulate their conservation status or false detection of pest species may come with wrong disease forecasting.

Although it is clear that the potential of eDNA-based monitoring in biological research is almost limitless, but scientific collaboration and coordination is still needed. The eDNA-based monitoring, especially eDNA metabarcoding, has the potential to bring together several fields from ecosystem restoration to human health. As the technology keeps updating and procedures optimizing, eDNA-based monitoring is likely to become an essential tool, extremely versatile, and essential for the future of molecular research.

## 7. Conclusions

The protection of species, habitats, and ecosystems from extinction, and destruction of biotic interactions, is the main issue for the conservation of nature and biodiversity. The accurate detection technique/method of micro- and macro-organisms (e.g., individuals, populations, community, etc.) in environmental samples (air, water, and soil) is essential for conservation management. As per molecular biologists and ecologists, the eDNA-based monitoring systems are applicable in conservation research (e.g., detection of invasive species and species under conservation) with high accuracy and authentication, due to effective approach (over 90% accuracy) in the monitoring and management of natural populations, compared to traditional surveys. However, until now, researchers are struggling with the application of proper eDNA technique due to lack of proper (i) research design, (ii) sampling, and (iii) sample-dependent methodology, etc., since the eDNA varies qualitatively and quantitively from one environment to another. The persistence of targeted eDNA depends on life history, biological properties, and physicochemical characteristics of the environment (e.g., temperature, pH, oxygen, conductivity, moisture content, light (visible/UV) exposure, nuclease activity, microbial activity, etc.). Thus, eDNA persistence and biology of target organisms are needed to properly design and optimize the sample-dependent (targeted species) and environment-dependent (e.g., aquatic, terrestrial, etc.) protocol, including preservation. The eDNA is subjected to amplification with species-specific primer for single species detection (barcoding), where qPCR is more effective compared to cPCR. However, in comparison to qPCR, the ddPCR exhibits more efficiency for species specificity and accurate quantification. In recent years, CRISPR-Cas is gaining popularity in eDNA-based species detection. The eDNA-based monitoring, especially eDNA metabarcoding, has the potential to bring together several fields; from ecosystem restoration to human health. Thus, the eDNA technique is significantly applicable in conservation biology, in the specific areas of early detection of invasive species, species detection for conservation, the community level biodiversity monitoring, ecosystem health monitoring, study on trophic interactions, etc. In addition, eDNA can be implemented to monitor the recently extinct species, as if they are still present in the wild, for this eDNA-based method is more suitable. However, a comprehensive eDNA-based monitoring program for the future management decisions, ecosystem modeling and function should be executed on a global scale. Forthwith, the governmental and academic-industrial collaborations are essential to make an eDNA survey toolkit for rapid, cost-effective, and non-invasive biodiversity monitoring.

## Figures and Tables

**Table 1:** Detection invasive species using eDNA, including species under study, target region and environment.

Taxonomic Group	Environment	Species and Target Region	Reference
Amphibian	Pond	*Lithobates catesbeianus* ^Cyt-b^	[8]
Angiosperms	River, Lake & Stream	*Elodea canadensis* ^trnL^	[56]
Arthropod	Freshwater sources	*Aedes albopictus* ^ITS^; *Ae. j. japonicus*^COI^, *Ae. Koreicus*^COI^	[57]
	Lake	*Eriocheir sinensis*, *Carcinus maenus*^COI^	[58]
		*Orconectes rusticus*; *Pacifastacus leniusculus*^COI^	[59]
	Seawater	*Rhithropanopeus harrisii* ^COI^	[60]
	River and Lake	*Crangonyx pseudogracilis* ^COI^	[61]
	Dust sample	Community ^COI^	[62]
Fish	Aquarium, River & Reservoirs	*Oncorhynchus mykiss*, *Salmo salar*, *Salmo trutta*, *Salvelinus fontinalis*, and *Salvelinus namaycush*^16s^	[63]
	Fresh water tank	*Oreochromis mossambicus* ^16s^	[64]
	River, Lake & Creek	*Esox lucius* ^COI, Cyt-b^	[65]
	Pond	*Lepomis macrochirus* ^Cyt-b^	[15]
	River & Reservoirs	*Cyprinus carpio* ^SNPs^	[66]
	River & Lake	*Hypophthalmichthys nobilis*, *H. molitrix*^MDL^	[9,54]
	Streams	*Salvelinus fontinalis* ^Cyt-b^	[67]
Invertebrate	Seawater	*Sabella spallanzanii* ^COI^	[68]
		*Bugula neritina* ^COI^	[69]
Mammal	Streams & Creek	*Sus scrofa* ^MDL^	[70]
Mollusca	Estuaries	*Xenostrobus securis* ^COI^	[71]
	River, Lake & Stream	*Dreissena polymorpha*, *D. bugensis*, *D. rostriformis Limnoperna fortune*^COI^; *Dreissena* sp. ^18s^	[58,72,73]
	Lakes	Community ^16s^	[74]
	Seawater	*Crepidula fornicata* ^COI^	[75]
		*Rangia cuneata* ^16s^	[76]
Reptile	Freshwater	*Python bivittatus* ^Cyt-b, ND4^	[34,53]
	Pond	*Trachemys scripta* ^COI^	[77]

N. B.: Mitochondrial Cytochrome c oxidase I: COI; Mitochondrial Cytochrome b: Cyt-b; rRNA 16s and 18s: ribosomal RNA 16s and 18s: 18s; Internal transcribed spacer: ITS; Mitochondrial NADH4: ND4; Mitochondrial D loop: MDL; Single nucleotide polymorphisms: SNPs; Chloroplast tRNA gene: trnL.

**Table 2:** eDNA studies used to assist conservation management, including target species, conservation status, target region and environment.

Taxonomic Group	Environment	Species, Conservation Status, Detection Method, Target Region	Reference
Amphibian	Pond	*Triturus cristatus* ^LC, qP, Cyt-b^	[80]
	Pool	*Pelophylax lessonae* ^LC, qP, Cyt-b^	[81]
	Stream	*Odorrana splendida* ^EN, qP, Cyt-b^	[82]
		*Onychodactylus japonicus* ^LC, qP, 12s^	[83]
	Drainage	*Cryptobranchus alleganiensis* ^NT, qP, Cyt-b^	[84]
	Stream	*Cryptobranchus alleganiensis alleganiensis* ^NT, qP, Cyt-b^	[45]
		*Hynobius vandenburghi* ^EN, qP, Cyt-b,12s^	[30]
	Bromeliads’ water	*Phytotriades auratus* ^EN, qP, Cyt-b^	[85]
Angiosperm	Rhizospheric soil/Flora	*Sapria himalayana* ^EN, qP, ITS^	[86]
Arthropod	Caves/springs (Water)	*Cambarus speleocoopi* ^EN, qP, COI^	[87]
	River/pond	*Austropotamobius pallipes* ^EN, qP, COI^	[88]
	River/Lake/Spring Creek	*Pacifastacus fortis* ^CR, qP, COI^	[89]
	River/lake	*Baetis buceratus* ^VU, cP, COI^	[61]
	Harbor	*Zearaja maugeana* ^EN, qP, ND4^	[90]
	River/lake	*Opsariichthys uncirostris uncirostris* ^Th, qP, MDL^	[91]
	River	*Pristis pristis* ^CR, cP, COI^	[78]
		*Plecoglossus altivelis ryukyuensis* ^EN, qP, ND4^	[92]
		*Oncorhynchus tshawytscha* ^EN, qP, COI^	[93]
Fish	River	*Spirinchus lanceolatus* ^Th, qP, Cyt-b^	[94]
	River/lake	*Hypophthalmichthys nobilis* ^DD^ *Hypophthalmichthys molitrix* ^NT, cP, MDL^	[9]
	Strems	*Salvelinus confluentus* ^VU, qP, Cyt-b^	[67]
	Wetland	*Misgurnus fossilis* ^LC, qP, Cyt-b, COI^	[95,96]
		*Acipenser medirostris* ^NT, qP, COI^	[97]
	Sea	*Kogia sima* ^DD, MB, 12s^	[98]
Heteropterans	Streams/wetland	*Nepa hoffmanni* ^EN, qP, 16s^	[99]
Mammals	Lake	*Neophocaena asiaeorientalis* ^EN, MB, 16s, Cyt-b^	[20,100]
Reptile	Streams	*Shinisaurus crocodilurus* ^EN, qP, Cyt-b^	[101]

N.B.: Mitochondrial Cytochrome c oxidase I: COI; Mitochondrial Cytochrome b: Cyt-b; rRNA 16s: 16s; rRNA 12s: 12s; Internal Transcribed Spacer: ITS; Mitochondrial NADH4: ND4; Mitochondrial D loop: MDL; qPCR: qP; cPCR: cP; Metabarcoding: MB; Least concern: LC; Endangered: EN; Near threatened: NT; Vulnerable: VU; Critically Endangered: CR; Threatened: Th; Data Deficient: DD.

**Table 3:** Biodiversity detection of aquatic and terrestrial environment in community level by eDNA technique.

Taxonomic Group	Environment and Target Region	Reference
Arthropod	Dust sample ^COI^	[62]
	Wild flower ^COI,^ ^16s^	[39]
Bird	Water from Zoo cages ^12s^	[50]
Eukaryote	Soil, scat, plant material & arthropods ^COI, 12s^	[109]
	Freshwater sediments ^18s^	[110]
	Freshwater ^COI^	[5]
	Seawater ^COI, 18s^	[111,112]
Fish	Seawater ^12s^	[113,114]
Fungi	Soil and organic litter ^COI, ITS, 18s^	[38]
Mammal	Forest pond water ^12s^	[115]
	Fly derived DNA ^16s^	[42]
Mollusc	Lake ^16s^	[74]
Plant and fungi	Air ^ITS^	[23]
Plant	Wetland ^18s, trnL^	[103]
Vertebrate	Bulk Arthropod ^12s, 16s^	[104]

N.B.: Mitochondrial Cytochrome c oxidase I: COI; rRNA 12s: 12s; rRNA 16s: 16s; rRNA 18s: 18s; Internal Transcribed Spacer: ITS; chloroplast tRNA gene: trnL.

## Data Availability

The study did not report any data.
